# B-Cell-Intrinsic Hepatitis C Virus Expression Leads to B-Cell-Lymphomagenesis and Induction of NF-κB Signalling

**DOI:** 10.1371/journal.pone.0091373

**Published:** 2014-03-20

**Authors:** Yuri Kasama, Takuo Mizukami, Hideki Kusunoki, Jan Peveling-Oberhag, Yasumasa Nishito, Makoto Ozawa, Michinori Kohara, Toshiaki Mizuochi, Kyoko Tsukiyama-Kohara

**Affiliations:** 1 Department of Experimental Phylaxiology, Faculty of Life Sciences, Kumamoto University, Kumamoto-shi, Kumamoto, Japan; 2 Department of Research on Blood and Biological Products, National Institute of Infectious Diseases, Musashi-Murayama-shi, Tokyo, Japan; 3 Department of Internal Medicine, Goethe-University Hospital, Frankfurt, Germany; 4 Center for Microarray Analysis, Tokyo Metropolitan Institute of Medical Science, Kamikitazawa, Tokyo, Japan; 5 Department of Microbiology and Cell Biology, Tokyo Metropolitan Institute of Medical Science, Kamikitazawa, Tokyo, Japan; 6 Transboundary Animal Diseases Center, Joint Faculty of Veterinary Medicine, Kagoshima University, Kagoshima, Japan; 7 Laboratory of Animal Hygiene, Joint Faculty of Veterinary Medicine, Kagoshima University, Kagoshima, Japan; Saint Louis University, United States of America

## Abstract

Hepatitis C virus (HCV) infection leads to the development of hepatic diseases, as well as extrahepatic disorders such as B-cell non-Hodgkin's lymphoma (B-NHL). To reveal the molecular signalling pathways responsible for HCV-associated B-NHL development, we utilised transgenic (Tg) mice that express the full-length HCV genome specifically in B cells and develop non-Hodgkin type B-cell lymphomas (BCLs). The gene expression profiles in B cells from BCL-developing HCV-Tg mice, from BCL-non-developing HCV-Tg mice, and from BCL-non-developing HCV-negative mice were analysed by genome-wide microarray. In BCLs from HCV-Tg mice, the expression of various genes was modified, and for some genes, expression was influenced by the gender of the animals. Markedly modified genes such as Fos, C3, LTβR, A20, NF-κB and miR-26b in BCLs were further characterised using specific assays. We propose that activation of both canonical and alternative NF-κB signalling pathways and down-regulation of miR-26b contribute to the development of HCV-associated B-NHL.

## Introduction

Approximately 200 million people are currently infected with the hepatitis C virus (HCV) worldwide [1]. HCV has been the major etiological agent of post-transfusion hepatitis and has frequently caused liver cirrhosis and hepatocellular carcinoma in chronic hepatitis C (CHC) patients [2], [3]. Hepatocytes are considered to be the primary and major site of HCV replication; however, extrahepatic manifestations are commonly seen in CHC patients. For example, mixed cryoglobulinemia (MC), a systemic immune complex-mediated disorder characterised by B cell proliferation with the risk of evolving into overt B-cell non-Hodgkin's lymphoma (B-NHL), is frequently recognised in CHC patients [4]–[6]. We have previously demonstrated the presence of both HCV RNA and viral proteins in peripheral B cells of CHC patients [7], although the mode of HCV infection and possible HCV replication in peripheral B cells remains a matter of debate. Furthermore, in the last two decades, an array of epidemiological evidence has accumulated involving the association between HCV infection and the occurrence of several hematologic malignancies, most notably B-NHL [8], [9]. The most compelling argument for a causal relationship between HCV and the occurrence of B-NHL is made by interventional studies demonstrating that a sustained virologic response to antiviral treatments, including the interferon α-induced regression of HCV-associated lymphomas and viral relapse after the initial virologic response, led to lymphoma recurrence [10]. However, the mechanisms underlying the cause-and-effect relationship are mostly unknown.

One of the potential host factors involved in HCV-associated B-NHL development is activator protein 1 (AP-1), which is primarily composed of c-Jun, c-Fos, and JunB, while JunD or Fra-1, Fra-2 and FosB are involved less frequently [11]. AP-1 is involved in B cell lymphomagenesis, is repressed by B cell lymphoma-6 [12] and is inhibited by the overexpression of T cell leukaemia/lymphoma 1, which resulted in the enhancement of nuclear factor kappa B (NF-κB) [13].

NF-κB is a ubiquitously expressed transcription factor that regulates a wide array of cellular processes, including the immune response, cell growth and differentiation [14], [15]. The activation of NF-κB is regulated by two distinct pathways termed the ‘canonical’ and the ‘alternative’ NF-κB signalling pathways. Representative stimulators of the canonical and alternative pathways are tumour necrosis factor αTNFα) and lymphotoxin α and β (LTα and LTβ), respectively [16]. Previous studies have demonstrated that NF-κB is activated via both the canonical [17], [18] and alternative [19] pathways in chronic HCV infection [17], [18] and HCV-related B-NHL [20]. However, the key NF-κB-activating pathway involved in HCV-associated B-NHL remains unknown.

TNFα-induced protein 3 (TNFAIP3), also known as A20, was first identified as a TNF-induced cytoplasmic protein with zinc finger motifs [21]. A20 has since been described as playing a pivotal role in the negative regulation of inflammation by terminating the canonical NF-κB signalling pathway [22]–[24]. Recently, A20 has gained attention as a novel tumour suppressor. For example, A20 was reported to be frequently inactivated or even deleted from mantle-cell lymphoma [25], [26] and diffuse large B-cell lymphoma (DLBCL) [27]. These findings raise the possibility that inactivation of A20 is, at least partially, responsible for lymphomagenesis [28]–[30]. Other investigators have subsequently confirmed these findings [27], [31]. Moreover, A20 also regulates antiviral signalling [32] as well as programmed cell death [33]–[35].

microRNAs (miRNAs) play a role in controlling various biological functions, including cell differentiation, growth regulation and transcriptional regulation [36]. In general, the dysfunctional expression of miRNAs is considered to be a common hallmark of cancers, including lymphomas [37]. HCV has been shown to influence miRNA expression *in vivo* and *in vitro* and utilises the liver-specific microRNA miR-122 for its replication [38]. The expression of miRNAs is also known to involve NF-κB activation. For example, miR-125a and miR-125b, both of which are often duplicated and/or overexpressed in DLBCL, were shown to activate NF-κB by targeting the A20 [39] and NF-κB-mediated dysregulation of miRNAs observed in lymphoma[40]. Moreover, global miRNA expression profiling analysis revealed miR-26b down-regulation in HCV-related splenic marginal zone lymphomas (SMZL) [41]. The same miRNA was found to be downregulated in peripheral blood mononuclear cells (PBMCs) from HCV-positive MC and NHL subjects [42].

We recently established transgenic mice that express the full-length HCV genome specifically in B cells (HCV-Tg mice) and observed the incidence of non-Hodgkin type B-cell lymphoma (BCL), primarily DLBCL, within 600 days after birth in approximately 25% of the HCV-Tg mice [43]. This experimental model is a useful tool for analysing the mechanisms underlying the development of HCV-associated manifestations such as B-NHL. To reveal the molecular signalling pathways responsible for HCV-associated B-NHL development, we performed a comprehensive molecular analysis of BCLs in HCV-Tg mice using a genome-wide microarray. We also characterised miR-26b expression in BCLs from HCV-Tg mice. Our results suggest that the activation of both canonical and alternative NF-κB pathways is involved in HCV-associated B-NHL development.

## Materials and Methods

### Ethics Statement

This study was carried out in strict accordance with both the Guidelines for Animal Experimentation of the Japanese Association for Laboratory Animal Science and the Guide for the Care and Use of Laboratory Animals of the National Institutes of Health. All experiment protocols were approved by the institutional review boards of the regional ethics committees of Kumamoto University (A22-136) and Kagoshima University (H24-008).

### Animal experiments

The full-length HCV genome (Rz) under the conditional Cre/*lox*P expression system [44] with mice expressing the Cre enzyme under the transcriptional control of the B lineage–restricted gene *CD19* [45] was established as RzCD19Cre mice [43]. Wild-type (WT), Rz, CD19Cre, RzCD19Cre mice (129/sv, BALB/c and C57BL/6J mixed background) were maintained in conventional animal housing under specific pathogen-free conditions. CD19Cre and RzCD19Cre mice were bred to be heterozygous for the *Cre* allele.

### Isolation of B cells and their RNAs

Mouse B cells were isolated using MACS^R^ beads (Milteny Biotec, Bergisch Gladbach, Germany) and anti-CD19 antibody (Beckton Dickinson, Franklin Lake, NJ). For FACS analysis, B and T cell populations were characterised using FITC-conjugated anti-B220 antibody (Milteny Biotec) and phycoerythrin (PE)-conjugated anti-CD3 antibody (Milteny Biotec) (Figure S1A). B cell purity was routinely over 95%. Total RNA was extracted from the B cells using the acid guanidine thiocyanate phenol chloroform method [44], [46]. The RNA integrity number was measured with an Agilent 2100 Bioanalyzer (Agilent Technologies, Santa Clara, CA), and samples with values over 8.0 were subjected to microarray analysis (Figure S1B).

### Microarray analysis

For microarray analysis, total RNAs were extracted, and RNA integrity was assessed using a Bioanalyzer (Agilent Technologies). cRNA targets were synthesised and hybridised with Whole Mouse Genome Microarray (G4846A; Agilent Technologies), in accordance with the manufacturer's instructions. More than 2-fold changes in gene expression were considered to be significant. Array data were analysed using MetaCore^™^ software (Thomson Reuters Co., New York, NY). The results of microarray analysis were registered in the Gene Expression Omnibus (GEO) database under the accession number GSE54722.

### Quantitative RT-PCR

cDNA was synthesised from 0.5 or 1 μg of total RNA with a Superscript II kit (Life Technologies, Carlsbad, CA). TaqMan gene expression assays were custom-designed and manufactured by Life Technologies. RNA expression was quantified using the ABI 7500 real-time PCR system (Life Technologies) or the CFX96 system (BioRad, Hercules, CA).

### Western blot analysis

Whole-cell proteins were extracted using RIPA buffer. Protein concentrations were determined using the BCA Protein assay Kit-Reducing Agent Compatible (Pierce Biotechnology, Rockford, IL). Samples (∼10 μg) were loaded onto 10% SDS acrylamide gels, and gels were then transferred to PVDF membranes (Merck Millipore, Darmstadt, Germany). Membranes were blocked using 5% (w/v) non-fat milk for approximately 1 hour at room temperature and were then sequentially probed with primary and secondary antibodies at 4°C overnight and at room temperature for approximately 1 hour, respectively.

As primary antibodies, anti-A20 antibody (sc-166692; Santa Cruz Biotech, Dallas, TX), anti-A20 antibody (SAB3500036; Sigma-Aldrich, St. Louis, MO), anti-C3 antibody (D-19; Santa Cruz Biotech), anti-Fos (sc-52; Santa Cruz Biotech), anti-c-Jun(N) (sc-45; Santa Cruz Biotech) and anti-GAPDH-HRP (sc-20357; Santa Cruz Biotech) antibodies were used. Secondary antibodies used were horseradish peroxidase-coupled donkey anti-rabbit Ig (NA934; GE Healthcare, Buckinghamshire, UK) and horseradish peroxidase-coupled sheep anti-mouse Ig (NA931; GE Healthcare). Protein bands were detected and quantified using either SuperSignal West Dura or Femto Extended Duration Substrate (Pierce Biotechnology) with a LAS-3000 Image Analyzer (Fuji Film, Tokyo, Japan). Stripping and re-probing of the Western blots were performed using Re-blot plus mild antibody stripping solution (Merck Millipore).

### Histological preparation

Liver, spleen, thymus and lymph nodes were harvested from HCV-Tg mice and fixed in 4% (wt/vol) paraformaldehyde in phosphate-buffered saline (pH 7.5) at 4°C for 24 hours. After fixation, samples were dehydrated in a graded ethanol series, cleared in xylene and embedded in paraffin, and 4-μm semi-thin sections were prepared using a carbon steel blade (Feather Safety Razor Co., Osaka, Japan) on a microtome (Yamato Kouki, Tokyo, Japan). Tissue sections were mounted on super-frosted glass slides coated with methyl-amino-silane (Matsunami Glass, Osaka, Japan). Histological images were acquired using an Olympus BX53 microscope (Olympus, Tokyo, Japan) equipped with 10×/0.30, 20×/0.50, 40×/0.75, and 100×/1.30 NA objective lenses. Images were captured using an Olympus DP73 (Olympus) under an Olympus FV1000 confocal microscope (Olympus).

### Immunofluorescence

Anti-mouse NF-κB p65 antibody (Ab7970; Abcam, Cambridge, UK) and anti-mouse B220 (14-0452-81; eBioscience, San Diego, CA) were used as primary antibodies, and donkey anti-rat IgG-Alexa Fluor 488 [712-545-153; Jackson ImmunoResearch Laboratories Inc. (JIR), West Grove, PA], donkey anti-rabbit IgG-Alexa Fluor 488 (711-545-152; JIR), donkey anti-rat IgG-Cy3 (712-165-153; JIR) and donkey anti-rabbit IgG-Cy3 (711-165-152; JIR) were used as secondary antibodies. Staining was conducted as described previously [47]. Briefly, antigen retrieval was performed in a steam pressure cooker with prewarmed antigen retrieval buffer, citrate pH 6 (S203130; Dako, Glostrup, Denmark) at 95°C for 15 min. After blocking with 3% bovine serum albumin in phosphate-buffered saline, sections (4 μm) were incubated with anti-NF-κB, -Iκ-B, -B200 or -A20 antibodies at a 1∶200 dilution each at 4°C overnight. Sections were incubated with secondary antibodies and anti-rat Alexa Fluor 488, -rabbit Alexa Fluor 488, -rat Alexa Fluor 546, and -rabbit Alexa Fluor 546 at room temperature for 2 hours. Nuclei were stained with Hoechst 333421 (H3570; Life Technologies).

### Single assay stem-loop Q-RT-PCR/ miR-26b analysis

Formalin-fixed, paraffin-embedded (FFPE) splenic tissue from 24 animals (BCL HCV+, n = 8; BCL HCV-, n = 5; non-tumorous spleen HCV+/−, n = 11) was selected for miR-26b expression analysis. Total RNA was extracted using an RNeasy FFPE Kit (Qiagen, Hilden, Germany) in accordance with the manufacturer's protocol. Single assay stem-loop Q-RT-PCR (TaqMan MicroRNA assays, Life Technologies) was used to quantify miRNAs in accordance with the manufacturer's protocol. Total RNA input for each reaction was 50 ng. Expression was analysed for hsa-miR-26b and an endogenous control (snoRNA202). Each sample was analysed in triplicate, and delta C_t_ values were calculated using endogenous controls.

### Statistics

For statistical analysis of NF-κB localisation, approximately 30–100 cells were randomly selected from each section area (two sections were used), and the cells double-positive for NF-κB and B220 were counted. All statistical analyses were performed using Prism software, version 5 (GraphPad, San Diego, CA). All experiments were independently performed three times, and a two-tailed Student *t*-test was applied to verify whether the results were significantly changed compared to the control (P<0.05).

## Results

### Characterisation of gene expression in B cells from HCV-Tg mice by microarray analysis

We previously established HCV-Tg mice that develop spontaneous BCL with a high penetrance (approximately 25%) [43]. To clarify the mechanisms of the HCV-associated B-NHL development using this mouse model, we performed a comprehensive gene expression analysis using a genome-wide microarray. B cells were isolated from BCL-developing HCV-Tg mice (Table 1, upper columns of pairing 1 and 3), from BCL-non-developing HCV-Tg mice (lower columns of pairing 1 and 3 and upper columns of pairing 2 and 4), and from BCL-non-developing HCV-negative mice (lower columns of pairing 2 and 4). RNA was purified from these B cells (Figure S1) and was characterised by microarray analysis (data not shown). In B cells isolated from BCL-non-developing HCV-Tg male mice, 455 and 863 genes were up- and down-regulated, respectively, compared with the HCV-negative counterparts (Table 1, pairing 2); whereas 133 and 331 genes were up- and down-regulated, respectively, in BCL-non-developing HCV-Tg female mice (Table 1, pairing 4). Furthermore, 1,682 and 2,383 genes were up- and down-regulated, respectively, in BCL-developing HCV-Tg male mice compared to their BCL-non-developing counterparts (Table 1, pairing 1); whereas 2,089 and 2,565 genes were up- and down-regulated, respectively, in BCL-developing HCV-Tg female mice (Table 1, pairing 3).

**Table 1:** Mice subjected to microarray analysis.

Pairing	Mouse genotype	Mouse (No)	Age (d)	Sex	Remarks
1	RzCD19Cre	24–1	748	male	HCV(+)BCL*
		59–1	723	male	
		69–5	710	male	
	RzCD19Cre	248–1	860	male	HCV(+) B cell
		288–3	472	male	
		299–1	385	male	
2	RzCD19Cre	307–2	212	male	HCV(+) B cell
		307–3	212	male	
	Rz, 4EBP(+/−)^+^	307–1	220	male	HCV(−) B cell
		312–1	220	male	
3	RzCD19Cre	54–1	724	female	HCV(+)BCL
		62–2	723	female	
	RzCD19Cre	308–4	219	female	HCV(+) B cell
		308–6	219	female	
4	RzCD19Cre	308–4	219	female	HCV(+) B cell
		308–6	219	female	
	Rz	308–1	219	female	HCV(−) B cell
		308–3	219	female	

*BCL: B cell lymphoma; ^+^4EBP(+/−): heterozygous knockout of 4E-BP1 gene [73].

### Metacore analysis of microarray results

**Figure 1:**
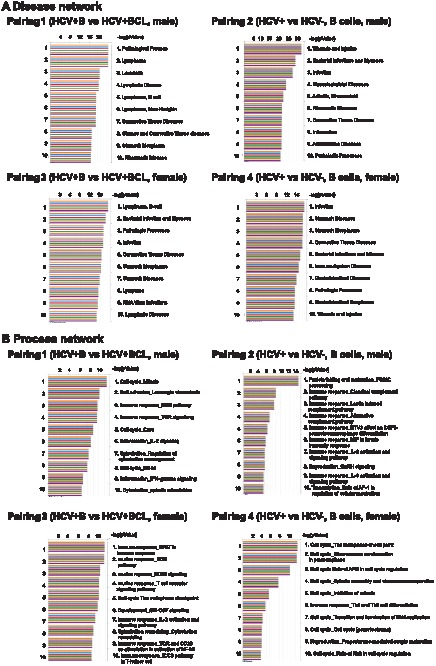
Data from array performed once with mixed RNA samples (**Table 1**) were analysed using MetaCore software. Signals were analysed in the disease network (**A**) and in the process network (**B**) the values for the microarray data [(Feature number; yellow), (Process Signal (635); blue), (Process signal (532); red), Test/Control (532/635); green], (Process Signal (635); orange), (Process signal (532); purple)] are indicated by coloured bars. Abbreviations: BCL = B cell lymphoma. Refer to Table 1 for construction of pairings.

In order to characterize the cellular processes affected by the gene expression changes, we carried out a pathway analysis of microarray data of pairings 1–4 (Table 1) using MetaCore^™^ software. This data mining revealed that lymphoma, leukaemia, B cell lymphoma, and lymphatic disease pathways were appreciably modified in pairings 1 and 3 with high frequency (Figure 1a). In pairings 2 and 4, the modifications involving wound healing and infection pathways were highlighted, respectively. In the process network, the cell cycle and immune response (B cell receptor, T cell receptor, and IL-2) pathways were greatly modified in pairings 1 and 3 (Figure 1b). The immune response (complement, macrophage, IL-2, and IL-3 in group 2; Th1 and Th2 in pairing), protein folding (in pairing 2), and cell cycle (in pairing 4) pathways were also modified.

### Dysregulated genes in HCV-associated B-cell lymphoma

**Figure 2:**
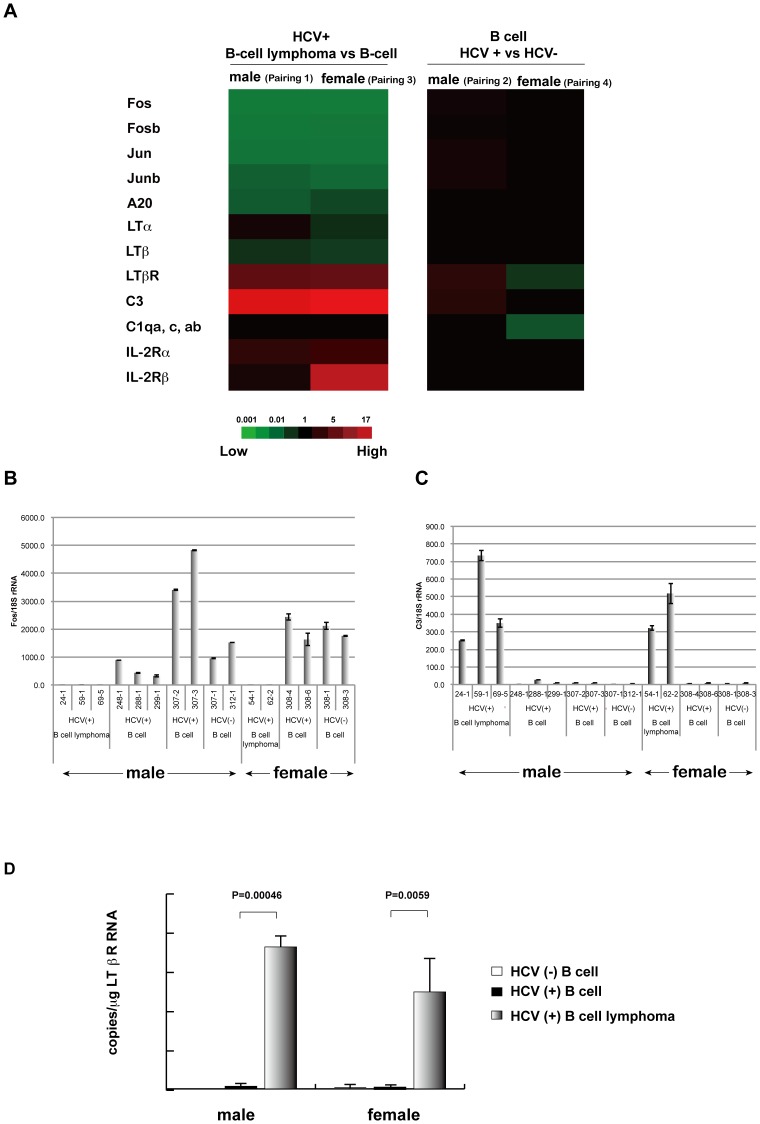
The expression of genes involved in oncogenic pathways associated with BCL. **A**: Highly modified gene signals in B cell lymphoma in RzCD19Cre mice BCL vs. B cells in RzCD19Cre male (Pair 1) or female (Pair 3) mice (left), and the genes modified by HCV expression in B cells in male (Pair 2) or female (Pair 4) (right). Red indicates the relative enhancement of the expression ratio of the processed signal (Test/Control, 532/635), and green indicates the relative reduction of expression. **B**: Quantification of Fos mRNA in HCV-, HCV+ B cells and HCV-Tg BCL in mice (numbers of individual mice were indicated) by quantitative RT-PCR. Fos mRNA was normalised against 18S rRNA, and the relative ratio was calculated. Vertical bars indicate S.D. **C**: Quantification of C3 mRNA in HCV-, HCV+ B cells and HCV-Tg BCL in mice. C3 mRNA was normalised against 18S rRNA, and relative ratio was calculated. Vertical bars indicate S.D. **D**: Quantification of LT βR mRNA in HCV-, HCV+ B cells and HCV-Tg BCL in mice by quantitative RT-PCR. RNA copies per total RNA (μg) were indicated and vertical bars indicate S.D.

In addition to the pathways analysis, we also carefully examined the expression of genes involved in oncogenic pathways associated with BCL. Expression of Fos, Fosb, Jun and Junb was markedly down-regulated in BCL obtained from HCV-Tg mice (Figure 2a). Similarly, the expression of A20 and LTβ was greatly down-regulated in BCL (Figure 2a). In contrast, the expression of the LTβ receptor (LTβR), the IL-2 receptor α(IL-2Rα, IL-2Rβ and complement C3 was up-regulated in the examined BCLs (Figure 2a). While alterations in the gene expression of LTα and IL-2Rβ differed between males and females, the overall mRNA expression profile in the BCL analysed from HCV-Tg mice essentially showed no differences between male and female mice. In addition, clinically, there was no clear gender priority in HCV-NHL [48]–[50]. These results suggest that the molecular signalling pathways leading to HCV-associated B-NHL development are common to males and females.

In non-tumorous B cells from BCL-non-developing HCV-Tg male mice, the expression of LTβR and C3 was up-regulated when compared with HCV-negative counterparts (Figure 2a). In contrast, in female counterparts, the expression of LTβR and complements C1qa, c, and ab was down-regulated (Figure 2a, Pair. 4). These results suggest that the impact of HCV infection in B cells may be different between males and females.

### Expression of Fos, C3, and LTβR genes in HCV-associated BCL

In order to validate the microarray results, levels of Fos and C3 mRNAs were quantified by real-time PCR. Striking down-regulation of Fos gene expression was observed in BCLs from HCV-Tg mice (Figure 2b). In contrast, C3 mRNA expression was markedly up-regulated in BCLs from HCV-Tg mice (Figure 2c). These results were consistent with the microarray data (Figure 2a, GEO accession number GSE54722). Similarly, the mRNA expression of the LTβR gene was significantly increased in HCV-associated BCLs (Figure 2d), confirming the microarray analysis results (Figure 2a). Importantly, these changes occurred in both male and female mice.

### Expression of A20 in HCV-associated BCL

**Figure 3:**
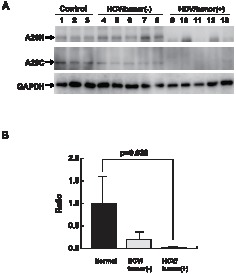
The expression of A20 in HCV-associated BCL. **A**: Expression levels of A20 in the spleen from RzCD19Cre mice with or without BCL. Whole-tissue extracts prepared from the spleen in CD19Cre mice (control, n = 3; lanes 1–3 217–2, 2 224–2, 224–3), RzCD19Cre mice without BCL (HCV/Tumour(-), n = 5; lanes 4–8 217–3, 224–4, 232–3, 254–4, 240–2) and RzCD19Cre mice with BCL (HCV/Tumour(+), n = 5; lanes 9–13 24–1, 56–5, 69–5, 59–1, 43–4) were subjected to SDS-PAGE and were analysed by immunoblotting using anti-N terminal (A20N), anti-C terminal A20 (A20C), and anti-GAPDH antibodies. GAPDH was used as protein loading control. **B**: Quantitation of A20 (N and C), the average is indicated and statistical analysis was performed. Vertical bars indicate S.D.

In order to further validate the microarray results, we assessed A20 protein levels in BCLs isolated from HCV-Tg mice by Western blotting (Figure 3a). Two distinct anti-A20 antibodies recognising the N- (A20N) and C-terminal regions were used for the detection of A20. Regardless of the anti-A20 antibodies used, expression levels of A20 in BCL from HCV-Tg mice (Figure 3a, lanes 9 to 13) were markedly decreased when compared to splenocytes obtained from either BCL-non-developing HCV-negative mice (lanes 1 to 3) or from BCL-non-developing HCV-Tg mice (lanes 4 to 8). Quantitative analysis showed a significant decrease in A20 in BCLs obtained from HCV-Tg mice (Figure 3b). These results strongly suggest that the reduced expression of A20 is correlated with HCV-associated N-BHL development.

### Nuclear localisation of NF-kB p65 in HCV-associated BCL

**Figure 4:**
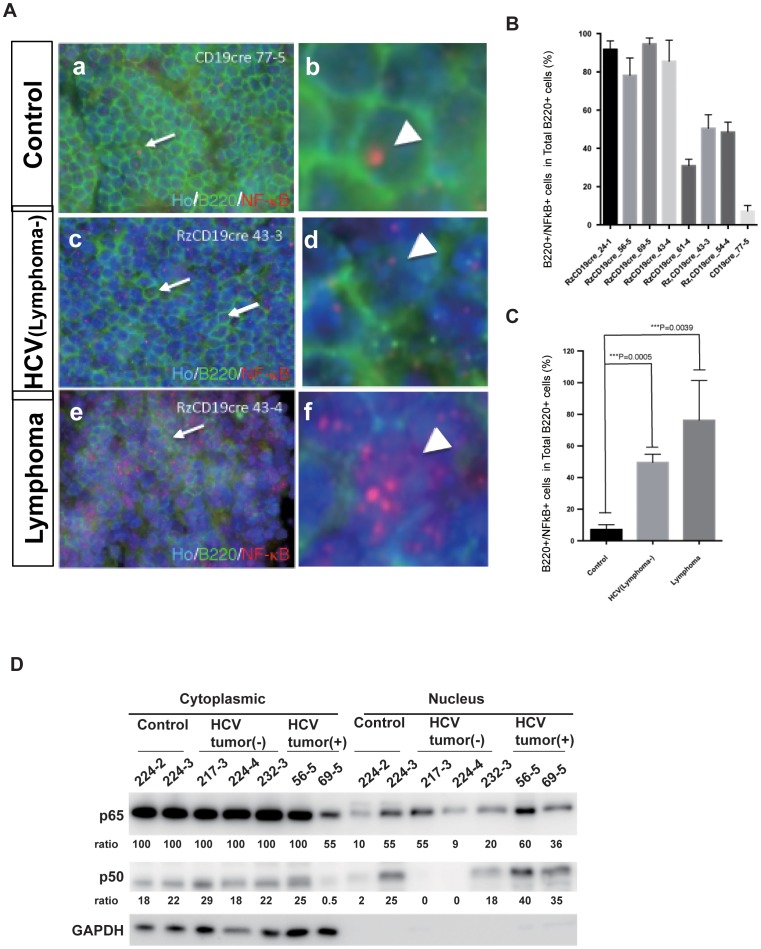
Double immunofluorescence localisation of B220 (Green) and NF-κB p65 (Red) in HCV-Tg mice and the fractionation analysis of mouse tissues. **A**: Co-localisation of NF-κB p65 immunoreactivity with B220 is indicated by arrows. (a–b) Cells double-positive for B220 and NF-κB in the control mouse (CD19cre). (c–d) Cells double-positive for B220 and NF-κB in the asymptomatic HCV-Tg mouse (RzCD19cre). (e–f) Cells double-positive for B220 and NF-κB in the lymphomatous HCV-Tg mouse (RzCD19cre). **B**: Quantitative analysis of the ratio of double-positive cells among B220-positive cells in each HCV-Tg mouse. Bar graph indicates the percentage of cells with NF-κB p65 nuclear translocation in B220-positive cells. **C**: Bar graph shows the ratio of double-positive cells within the B220-positive cells in normal, asymptomatic and lymphomatous HCV-Tg mice. Ho: Hoechst33342 Data are presented as means ± S.E., * P<0.05, ** P<0.01, *** P<0.001. **D**: Western blot analysis: tissues from the spleen of controls (224–2, 3) or HCV-Tg mice without BCL (217–3, 224–4, 232–3) or with BCL (56–5, 69–5) were fractionated into nuclear and cytoplasmic fractions. NF-κB p50 and p65 were detected by antibodies. Relative ratios of quantitation by imager are indicated. GAPDH was detected as a loading control of the cytoplasmic fraction.

We next analysed the activation status of NF-κB by investigating the nuclear localisation of NF-κB p65 in cells positive for a B-cell marker molecule, B220, in BCLs isolated from HCV-Tg mice (Figure 4a). Quantitative analysis revealed that the ratio of cells double-positive for B220 and NF-κB p65 in the nuclei of the examined BCLs was significantly higher than the ratio in splenic tissue obtained from either BCL-non-developing HCV-negative mice or from BCL-non-developing HCV-Tg mice (Figures 4b and c). The fractionation assay showed that more NF-κB p50 and p65 were present in BCLs from HCV-Tg mice (Figure 4d). These results indicate the activation of NF-κB in HCV-associated BCL.

### Expression of miR-26b in HCV-associated BCL

**Figure 5:**
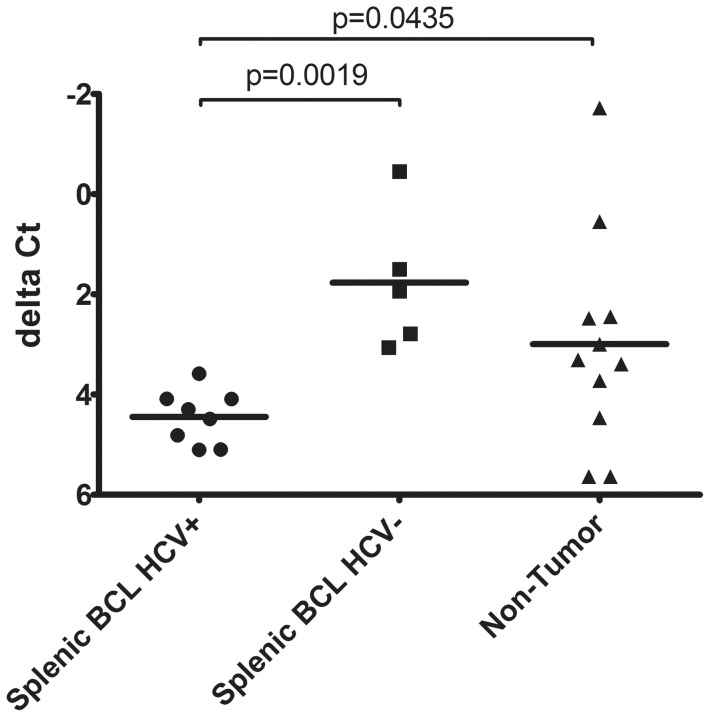
Quantification of miR-26b in BCL from HCV-positive and HCV-negative and non-tumour Tg mice. Formalin-fixed, paraffin-embedded (FFPE) splenic tissue from 24 animals (BCL HCV+, n = 8; BCL HCV-, n = 5; non-tumorous spleen HCV+/−, n = 11) was analysed for miR-26b expression by single assay stem-loop Q-RT-PCT by triplicate experiments. Data are shown as scatter dot-plots, and horizontal bar depicts the mean; y-axis: delta Ct (inverted scale) calculated in relation to endogenous control (snoRNA202). HCV-positive lymphoma tissue: filled circles; HCV-negative lymphoma tissue: filled squares; non-tumorous splenic tissue: filled triangles. P-values are shown in the graph.

Recent studies have demonstrated that miR-26b is down-regulated in hepatocellular carcinoma [51], nasopharyngeal carcinoma [52], primary squamous cell lung carcinoma [53] and squamous cell carcinoma of the tongue [54]. In addition, miR-26b was down-regulated in HCV-positive SMZL when compared with HCV-negative counterparts [41] and in the PBMC of HCV-positive MC and NHL patients [42]. Therefore, we compared the expression levels of miR-26b in BCL from HCV-Tg mice with BCL from HCV-negative mice (i.e., spontaneously developed BCL) or in splenic tissue from BCL non-developing HCV-positive and -negative mice (Figure. 5). Interestingly, miR-26b expression was significantly down-regulated in BCLs from HCV-Tg mice. These results indicate that miR-26b is also down-regulated in HCV-associated BCL.

## Discussion

In the present study, we identified differentially expressed genes in BCLs examined from HCV-Tg mice using a genome-wide microarray (Figures 1 and 2a, Table 1, and Figure S2). The microarray results for representative genes were validated at the RNA (Figures 2 and 5) and protein (Figures 3 and 4) levels. These findings helped dissect the molecular mechanisms underlying HCV-associated B-NHL development.

In the BCLs from HCV-Tg mice, the marked down-regulation of the Fos gene as well as other AP-1 protein genes (Fosb, Jun and Junb) was observed. Although AP-1 DNA binding activity was observed in Hodgkin-/multinuclear Reed-Stemberg cells and tissues from classical Hodgkin's disease, non-Hodgkin cell lines lacked the DNA binding activity of AP-1 [55]. Junb was weakly expressed in non-Hodgkin lymphomas of B-lymphoid origin; however, strong expression has been previously found in lymphomas that originated from the T-lymphoid lineage, and Junb selectively blocked B-lymphoid but not T-lymphoid cell proliferation ex vivo [56]. The BCL that developed in HCV-Tg mice was the non-Hodgkin type [43]; therefore, the decrease in AP-1 protein levels (Fos, Fosb, Jun, and Junb) may be crucial for lymphoma development.

In our previous study, soluble IL-2Rα levels were increased in BCL-developing HCV-Tg mice [43] Therefore, the up-regulation of IL-2Rα(Figure 2a) is potentially linked to the increase of soluble IL-2Rα, although further investigation is needed to clarify the details of this mechanism.

Expression of complement component C3 was significantly increased in BCLs isolated from HCV-Tg mice (Figure 2c). The presence of polymorphisms in complement system genes in non-Hodgkin lymphoma [57] suggests the involvement of complement in lymphoma development. The elevated C3 expression may be induced by TNF-α [58]. In addition, C3a, which is a cleavage product of C3, may contribute to the binding of NF-κB and AP-1 as shown previously [59].

The expression of LTβR, which is one of the key molecules in the alternative NF-κB signalling pathway [16], was significantly increased in BCLs from HCV-Tg mice (Figure 2d). HCV core proteins were reported to interact with the cytoplasmic domain of LTβR [60], [61] and to enhance the alternative NF-κB signalling pathway [62]. The induction of LTβR by the HCV non-structural protein NS5B, and HCV RNA-dependent RNA polymerase, was also observed [63]. These findings suggest that the regulatory pathways involved in HCV infection also play a role in HCV-associated B-NHL development.

We observed several differences in the gene expression between male and female mice. Male HCV-negative mice showed up-regulation of LTβR and C3; however, female HCV-positive mice featured the downregulation of LTα and up-regulation of IL-2Rβ. Female HCV-Tg mice showed decreased overall survival in a previous study [43] and the above-mentioned gene dysregulations may contribute to this finding. However, the incidence of B-NHL between male and female mice did not show marked differences in the transgenic model [43]. Some clinical studies found gender-specific differences in the incidence of HCV-associated B-NHL and different effects of HCV on gene expression, which may also be dependent on gender [64]. However, meta-analyses did not provide consistent evidence for any gender preferences in HCV-NHL [48]–[50].

The down-regulation of A20, which is a ubiquitin-editing enzyme and tumour suppressor in various lymphomas [26], was observed in BCLs from HCV-Tg mice (Figures 3a and 3b). A20 has been reported to interact with the TNF receptor associated factor 2 (TRAF2), TRAF6, and the NF-κB essential modulator (NEMO). A20 inhibits NF-κB activation-induced by TNFα or by the overexpression of other proteins such as TRAF2 and receptor-interacting protein serine/threonine kinase 1 (RIPK1) proteins [65]. RIPK3 contributes to TNFR1-mediated RIPK1-dependent apoptosis and necroptosis [66]. RIPK2 (also known as RIP2) is also involved in B cell lymphoma cell survival and mediates the activation of NF-κB and MAPK pathways, associated with the TNF receptor family [67]. Therefore, suppression of A20 activates NF-κB by increasing nuclear translocation in tumour tissues.

Expression of miR-26b in BCLs obtained from HCV-Tg mice was significantly down-regulated (Figure 5). miR-26b is also down-regulated in numerous cancers, e.g., HCC [51], nasopharyngeal carcinomas [52], primary squamous cell lung carcinomas [53]and squamous cell carcinoma tongue [54]. In addition, c-Myc, which is up-regulated in various cancer types, has been shown to contribute to the reduction of miR-26a/b expression [68]. Notably, expression of miR-26b was significantly down-regulated in SMZL arising in HCV-positive patients [41]. Although the mechanisms of miR-26b-mediated tumourigenicity regulation are not fully understood, previous reports [69] and the present study have suggested a possible regulatory role of miR-26b in HCV-related lymphoma. Several candidates are reported to be targets of miR-26b. miR-26a and miR-26b are regulators of EZH2, which is the PRC2 polycomb repressive complex, is overexpressed in multiple cancers and is a target of the MYC oncogene [70]. In addition, lymphoid enhancer factor (LEF)-1 [42] and Nek6 [41] are targets of miR-26b. LEF-1 is a nuclear transcription factor that forms a complex with β-catenine and T-cell factor and induces transcription of cyclin D1 and c-myc. Nek6 is a kinase involved in the initiation of mitosis and is overexpressed in various tumours. The phosphatase and tensin homolog gene (PTEN) is also the putative target gene of miR-26b in adipogenic regulation [71] and cell growth [72].

This report is the first to demonstrate the possible involvement of networks of NF-κB, AP-1, complements and miR-26b in HCV-associated B-NHL (Figure S2). A future study focusing on the dysregulation of these networks and their modification by HCV may provide valuable information on improving therapy for HCV-associated B-NHL.

## Supporting Information

Figure S1 **A**: B cells were isolated from mice using MACS beads and anti-CD19 antibody. The population of B cells was confirmed by staining with anti-B220 antibody. **B**: RNA integrity number (RIN) was measured using an Agilent 2100 Bioanalyzer (Agilent) for the estimation of purity.
(PDF)

Figure S2 **Possible pathways involved in BCL development. Both canonical and alternative NF-κB pathways may play a role.** Bold arrows indicate up-regulation or down-regulation. NIK; NF-κB-inducing kinase.
(PDF)
