# Supplementary figures and images for: B-Cell-Intrinsic Hepatitis C Virus Expression Leads to B-Cell-Lymphomagenesis and Induction of NF-κB Signalling
Source: PLoS One. 2014 Mar 20;9(3):e91373. doi: 10.1371/journal.pone.0091373 (PMC3961254; doi:10.1371/journal.pone.0091373)

**A**

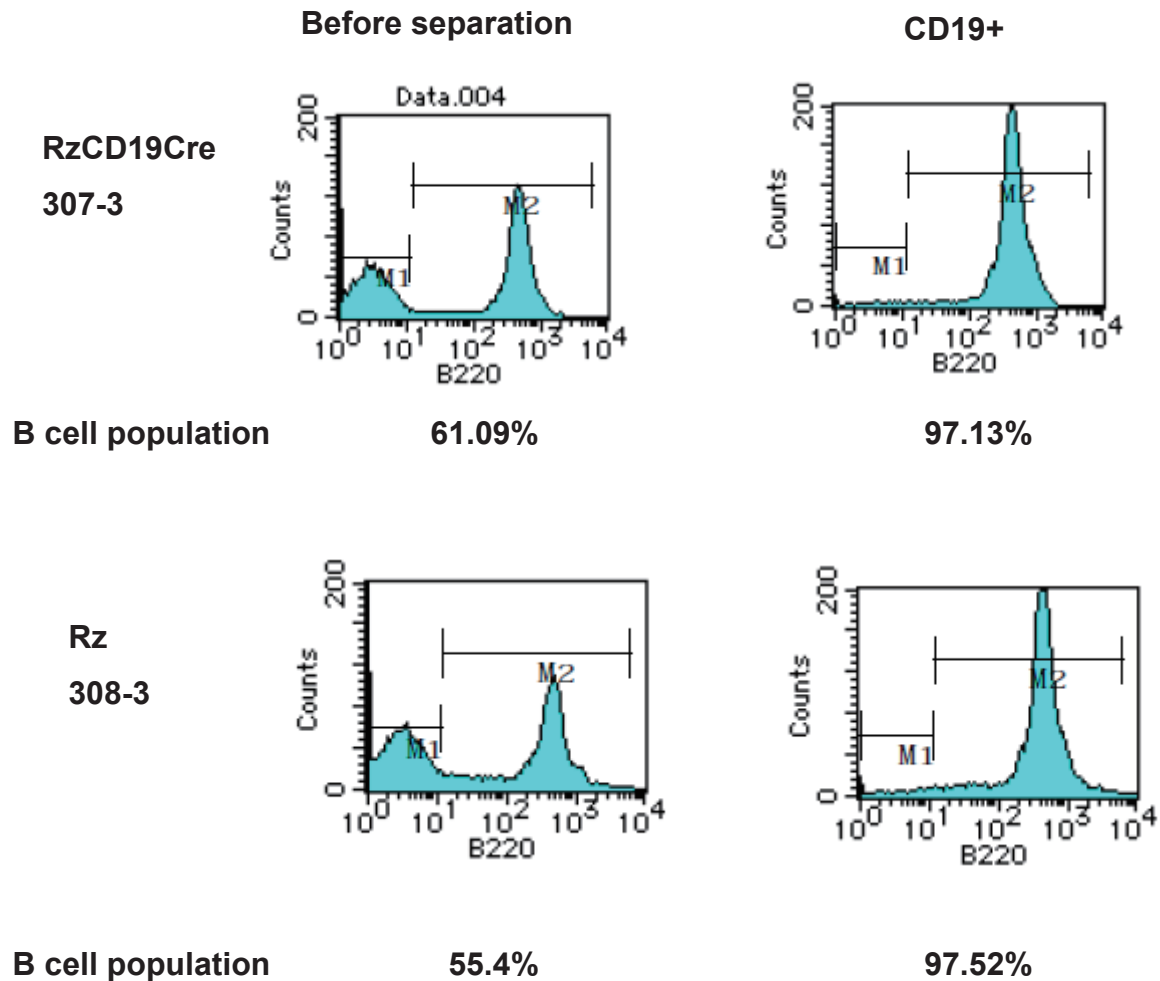

**B**

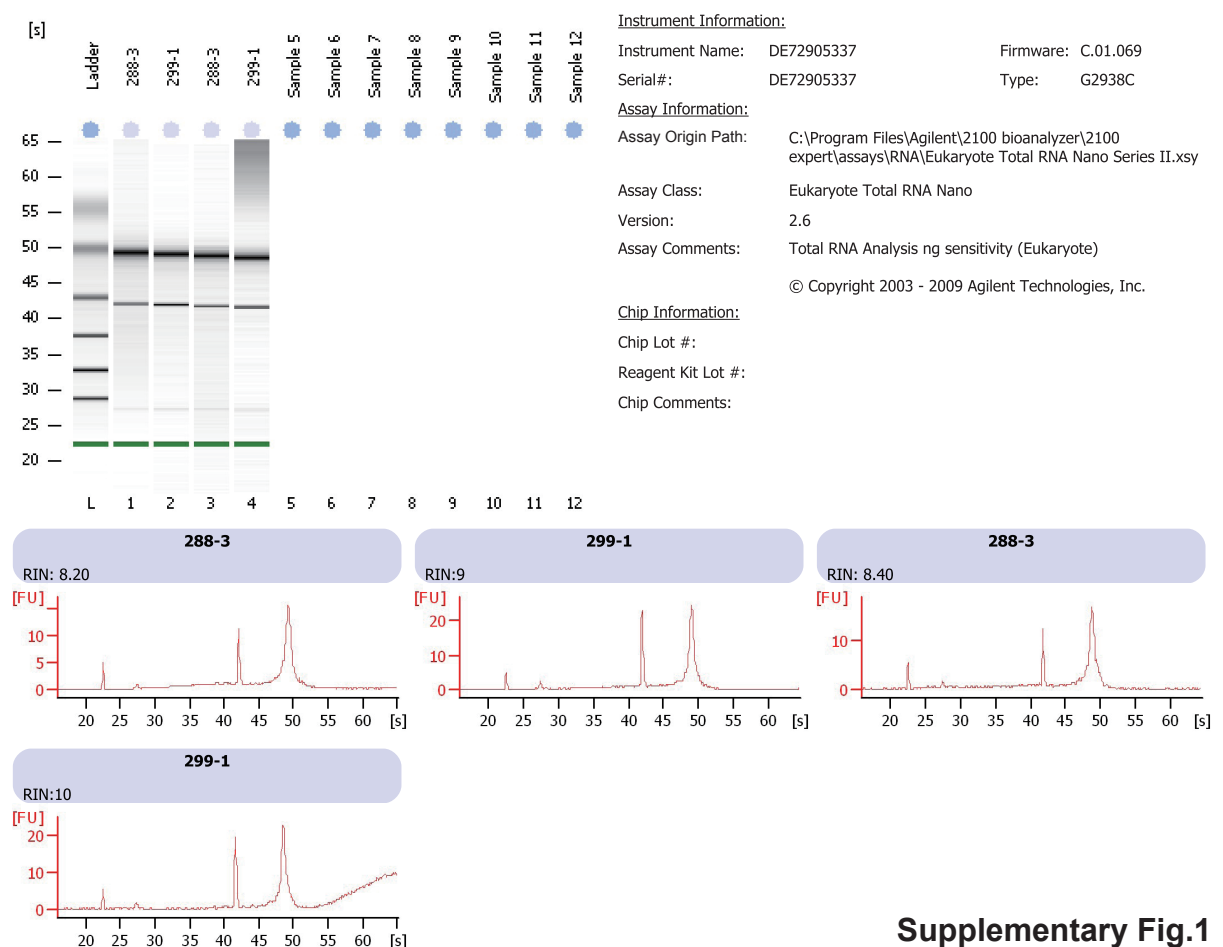

**Supplementary Fig.1**

Supplement: Figure S1 — A: B cells were isolated from mice using MACS beads and anti-CD19 antibody. The population of B cells was confirmed by staining with anti-B220 antibody. B: RNA integrity number (RIN) was measured using an Agilent 2100 Bioanalyzer (Agilent) for the estimation of purity. (PDF) [file pone.0091373.s001.pdf]

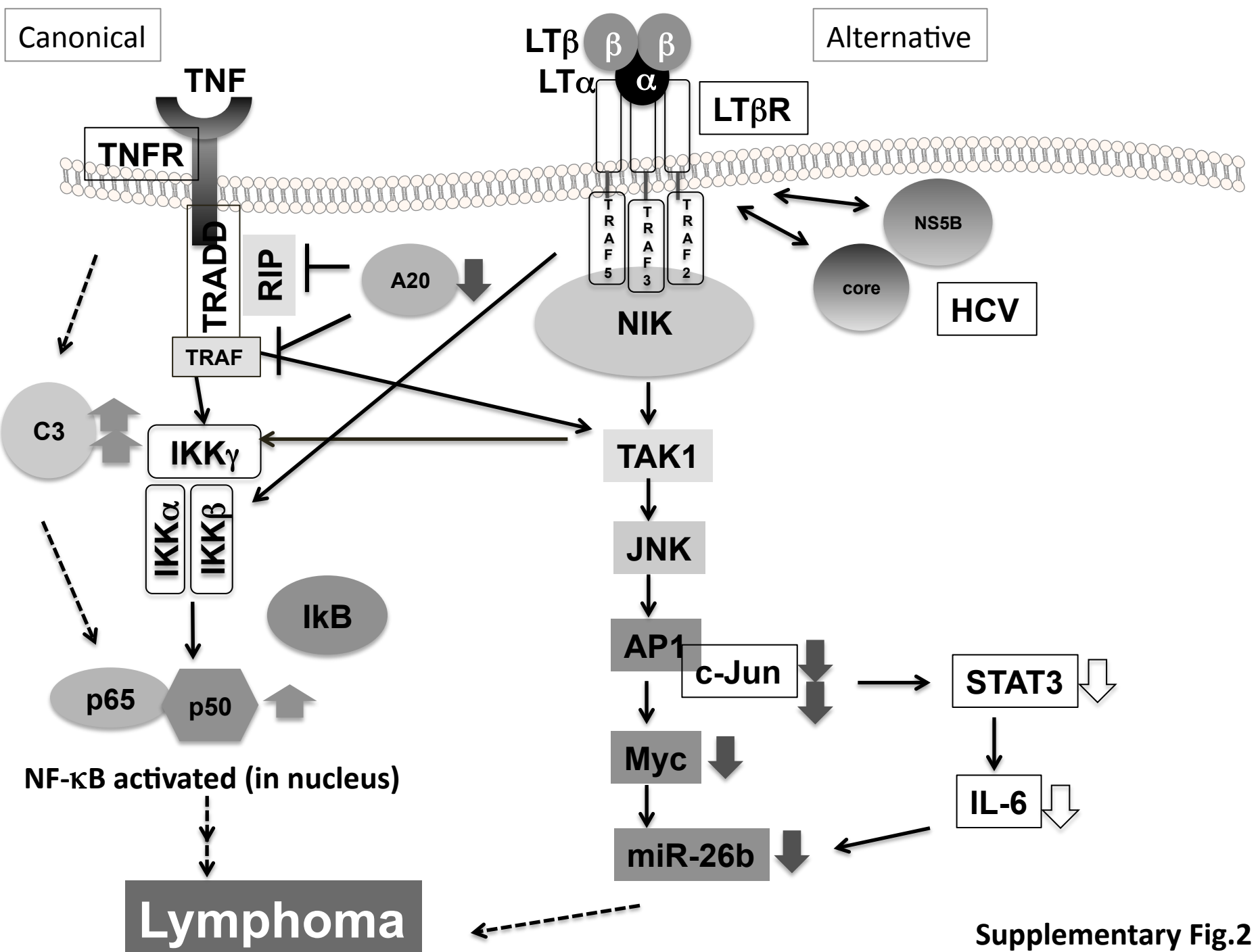

Supplementary Fig.2

Supplement: Figure S2 — Possible pathways involved in BCL development. Both canonical and alternative NF-κB pathways may play a role. Bold arrows indicate up-regulation or down-regulation. NIK; NF-κB-inducing kinase. (PDF) [file pone.0091373.s002.pdf]
